# Differential Effect of Cytomegalovirus Infection with Age on the Expression of CD57, CD300a, and CD161 on T-Cell Subpopulations

**DOI:** 10.3389/fimmu.2017.00649

**Published:** 2017-06-02

**Authors:** Fakhri Hassouneh, Nelson Lopez-Sejas, Carmen Campos, Beatriz Sanchez-Correa, Raquel Tarazona, Rafael Solana, Alejandra Pera

**Affiliations:** ^1^Maimonides Biomedicine Institute of Cordoba (IMIBIC), Reina Sofia Hospital, University of Cordoba, Cordoba, Spain; ^2^Immunology Unit, Department of Physiology, University of Extremadura, Cáceres, Spain; ^3^Division of Clinical and Experimental Medicine, Brighton and Sussex Medical School, Brighton, United Kingdom

**Keywords:** CD57, CD300a, CD161, T-cell subsets, age and cytomegalovirus infection

## Abstract

Immunosenescence is a progressive deterioration of the immune system with aging. It affects both innate and adaptive immunity limiting the response to pathogens and to vaccines. As chronic cytomegalovirus (CMV) infection is probably one of the major driving forces of immunosenescence, and its persistent infection results in functional and phenotypic changes to the T-cell repertoire, the aim of this study was to analyze the effect of CMV-seropositivity and aging on the expression of CD300a and CD161 inhibitory receptors, along with the expression of CD57 marker on CD4^+^, CD8^+^, CD8^+^CD56^+^ (NKT-Like) and CD4^−^CD8^−^ (DN) T-cell subsets. Our results showed that, regardless of the T-cell subset, CD57^−^CD161^−^CD300a^+^ T-cells expand with age in CMV-seropositive individuals, whereas CD57^−^CD161^+^CD300a^+^ T-cells decrease. Similarly, CD57^+^CD161^−^CD300a^+^ T-cells expand with age in CMV-seropositive individuals in all subsets except in DN cells and CD57^−^CD161^+^CD300a^−^ T-cells decrease in all T-cell subsets except in CD4^+^ T-cells. Besides, in young individuals, CMV latent infection associates with the expansion of CD57^+^CD161^−^CD300a^+^CD4^+^, CD57^−^CD161^−^CD300a^+^CD4^+^, CD57^+^CD161^−^CD300a^+^CD8^+^, CD57^−^CD161^−^CD300a^+^CD8^+^, CD57^+^CD161^−^CD300a^+^NKT-like, and CD57^+^CD161^−^CD300a^+^DN T-cells. Moreover, in young individuals, CD161 expression on T-cells is not affected by CMV infection. Changes of CD161 expression were only associated with age in the context of CMV latent infection. Besides, CD300a^+^CD57^+^CD161^+^ and CD300a^−^CD57^+^CD161^+^ phenotypes were not found in any of the T-cell subsets studied except in the DN subpopulation, indicating that in the majority of T-cells, CD161 and CD57 do not co-express. Thus, our results show that CMV latent infection impact on the immune system depends on the age of the individual, highlighting the importance of including CMV serology in any study regarding immunosenescence.

## Introduction

The human CD300 family has seven members, including the inhibitory receptor CD300a, which has been proposed as a possible biomarker for diagnosis and therapeutic target in several pathological situations (i.e., infectious diseases and cancer) (1–4). Human CD300a receptor is expressed on the surface of T (5, 6) and natural killer (NK) cells (7, 8). On human NK cells, the interaction between CD300a and its ligand reduces their cytotoxic function (8). In T and B cells, the primary function of CD300a is to limit antigen receptor-mediated positive signaling (9). However, on CD8^+^ T-cells, CD300a expression has been shown to associate with better cytotoxic function (10) and CD300a^+^CD4^+^ T-cells are associated with polyfunctionality and, upon stimulation, upregulate the transcription factor Eomesodermin (Eomes) (6, 11).

CD161 marker is a C-type lectin that was originally described in NK cells (12, 13). Nevertheless, CD161 is also expressed by T-cells including CD4^+^, CD8^+^ (12), and γδ T-cells (14). Within the CD4^+^ subset, CD161 expression has been associated with IL-17 production. Indeed, Th17 cells can be originated from the CD161^+^CD4^+^ but not from their CD161^−^CD4^+^ counterpart (15). Of note, other IL-17-producing T-cells, such as CD8^+^ and CD4^−^CD8^−^ double-negative T-cells are as well CD161^+^ (16). Furthermore, it has been shown that CD161 expression on T-cells characterizes a transcriptional and functional T-cell phenotype that is TCR- and cell lineage-independent (17). All CD161^+^ T-cell subsets shared a transcriptional signature and responded in a TCR-independent (innate-like) way to cytokine stimulation (IL-12 plus IL-18). However, CD161 had no regulatory effect on this response. Instead, CD161 has been shown to function as a costimulatory receptor in the context of TCR stimulation (18, 19). While the role of CD161 receptor on NK cells is agreed to be inhibitory (12, 20, 21), on T-cells, there is lack of consensus, as there is evidence of both costimulatory (20, 22) and inhibitory (18, 19) effects.

During aging, both innate and adaptive immunity are affected. Age-related changes have been described in several immune cell types including T-cells, NK cells, B-cells, macrophages, etc. Among those changes, the alterations in the number, phenotype, and functional capacity of immune cells have been associated with higher susceptibility to infectious diseases that ultimately lead to increased risk of fragility and death in those individuals (23–26). This age-associated deterioration of the immune system has been termed “immunosenescence.” However, immunosenescence is not exclusively due to chronological aging of the individual and there are situations involving chronic stimulation of the immune system, such as viral infections, in which an “immunosenescence accelerated” or “early immunosenescence” is observed (27–30). In humans, infection by a common virus, cytomegalovirus (HCMV) has been shown to have profound impact on the T-cell compartment both on CD8^+^ and CD4^+^ T-cells (31, 32). HCMV persists after primary infection and is continuously controlled by the immune system (33, 34). Human herpes viruses, like CMV, have generally a benign/symbiotic relationship with the host (35–38). However, this benign relationship between herpesviruses and its hosts is altered with age. Indeed, CMV latent infection has been related to early immunosenescence (32, 39, 40). Particularly, CMV-seropositivity is associated with an increased risk of death and cardiovascular diseases (41–43) and is a contributor to the development of an "Immune Risk Phenotype" (IRP). This IRP is associated with early mortality in the elderly (44–46). Therefore, HCMV is considered one of the most relevant contributors to immunosenescence.

Thus, both HCMV infection and age contribute to the process of immunosenescence inducing changes on the T-cells. Understanding the mechanisms leading to immunosenescence and finding new biomarkers could open the possibility of novel therapies for the treatment of age-related diseases. In that regard, here, we study the effect of CMV latent infection and age on the expression of CD161 and CD300a receptors on CD4^+^, CD8^+^, CD8^+^CD56^+^ (NKT-like), and CD4^−^CD8^−^ (DN) T-cell subsets and their relation with the polyfunctionality marker CD57, which is a hallmark of CMV infection and aging in T-cells (37, 38).

## Materials and Methods

### Subjects

We studied 64 healthy individuals stratified according to age and CMV serostatus (Table 1). Individuals in the old group and middle age group were all CMV-seropositive, as we were not able to recruit enough CMV-seronegative individuals due to the high prevalence of CMV seropositivity in Spain, which is about 80% in individuals over the age of 40 years (47) and reaches about 99% in individuals over 65 years in Andalusia (Southern Spain) where the samples were collected.

**Table 1:** Demographics of studied individuals (*n* = 64).

CMV	Age (years)	No.	Group name
Negative	18–35	22	Young CMV-seronegative
Positive	18–35	15	Young CMV-seropositive
Positive	40–65	13	Middle age CMV-serpositive
Positive	>70	14	Old CMV-serpositive

All subjects studied met the following exclusion criteria: absence of diabetes, cancer, severe renal failure, severe liver disease, endocrine disorders, autoimmune diseases, or acute infectious disease; they were not consuming drugs whose activity is known to modify the functions of the immune system. Ethical statement was approved by the Ethics Committee of the Reina Sofia University Hospital and all study participants provided informed written consent.

### CMV Serology

CMV-specific IgG and IgM was determined in sera by using automated enzyme-linked immunosorbent assay (ELISA) (DRG International, Mountainside, NY, USA).

### Flow Cytometry and Data Analysis

Peripheral blood from each subject was collected in lithium heparin tubes, followed by PBMCs isolation by density gradient centrifugation using Ficoll Histopage-1077 (Sigma-Aldrich, St. Louis, MO, USA). After isolation, PBMCs were cryopreserved until experiments were performed.

Cell thawing was carried out in RPMI 1640 (Sigma-Aldrich) with 10% FBS (Gibco, Life Technologies CA, USA) and cells were placed in a 96-well plate at 2 × 10^6^ cells/ml concentration (250 µl final volume). Subsequently, cells were washed twice with PBS (4°C) and stained for the following antibodies: anti-CD3 PerCP (clone: BW 264/56, MiltenyiBiotec), anti-CD56 phosphatidylethanolamine (PE)-Cy7 (clone: B159, BD Pharmingen), anti-CD57 VioBlue (clone: TB03, MiltenyiBiotec), anti-CD300a PE (clone: E59.126, Beckman Coulter), anti-CD4 FITC (clone: M-T466, MiltenyiBiotec), anti-CD8 APC-Cy7 (clone: SK1, BD Biosciences), and anti-CD161 APC (clone: DX12, BD Pharmingen). All antibodies were titrated before use.

Samples were acquired with a nine parameters MACsQuant instrument (Miltenyi Biotech, BergischGladbach, Germany) and analyzed with FlowJo v X 10.0.7 software (TreeStar, Portland, OR, USA). First, lymphocytes were gated according to their size and granularity (FSC vs SSC), then forward scatter height versus forward scatter area to remove doublets. Within that gate (singlets), CD3^+^ T-cells were gated, followed by identification of the different T-cell subsets by confronting CD4 vs CD8. NKT-like cells (CD8^+^CD56^+^) were then gated from CD8^+^ T-cells (Figure S1A in Supplementary Material). The average number of events acquired for each subset was: 71161 cells for CD4^+^ subset, 32498 cells for CD8^+^, 5708 cells for NKT-like, and 5511 cells for DN. Individual gates (set based on fluorescence minus one controls) for CD57^+^, CD161^+^, and CD300^+^ cells were gated on each of these populations (Figure S1B in Supplementary Material). FlowJo’s Boolean gating options were performed to analyze the co-expression of CD57, CD161, and CD300a markers.

### Statistical Analysis

Data were inspected for normal distribution using the Shapiro–Wilk test. No normality was found. According to this, Kruskal–Wallis *H* test (non-parametric test) with correction for multiple comparisons was used for direct comparison of the four groups. Those variables in which we found a statistical significant difference were then analyzed using the Mann–Whitney *U* non-parametric test for comparing data among the specific sample pairs. All statistical tests were performed with PASW Statistics v18. For scatter graphs, GraphPad Prism (version 5.0) was used. All graphs reflect only the Mann–Whitney derived *p*-values. To compare the pie charts, we used SPICE’s permutation analysis (Mario Roederer, ImmunoTechnology Section, Vaccine Research Centre, NIH, Bethesda, MD, USA) (48), which asks how often given the samples that comprise the two pies charts, the difference observed would happen simply by chance (10,000 permutations).

## Results

### CD57, CD161, and CD300a Expression on T-Cells

Multicolor flow cytometry was used to analyze the expression of CD57, CD161, and CD300a markers on CD4^+^, CD8^+^, NKT-like, and DN T-cell subpopulations from healthy individuals stratified by age and CMV-serostatus (Table 1).

**Figure 1:**
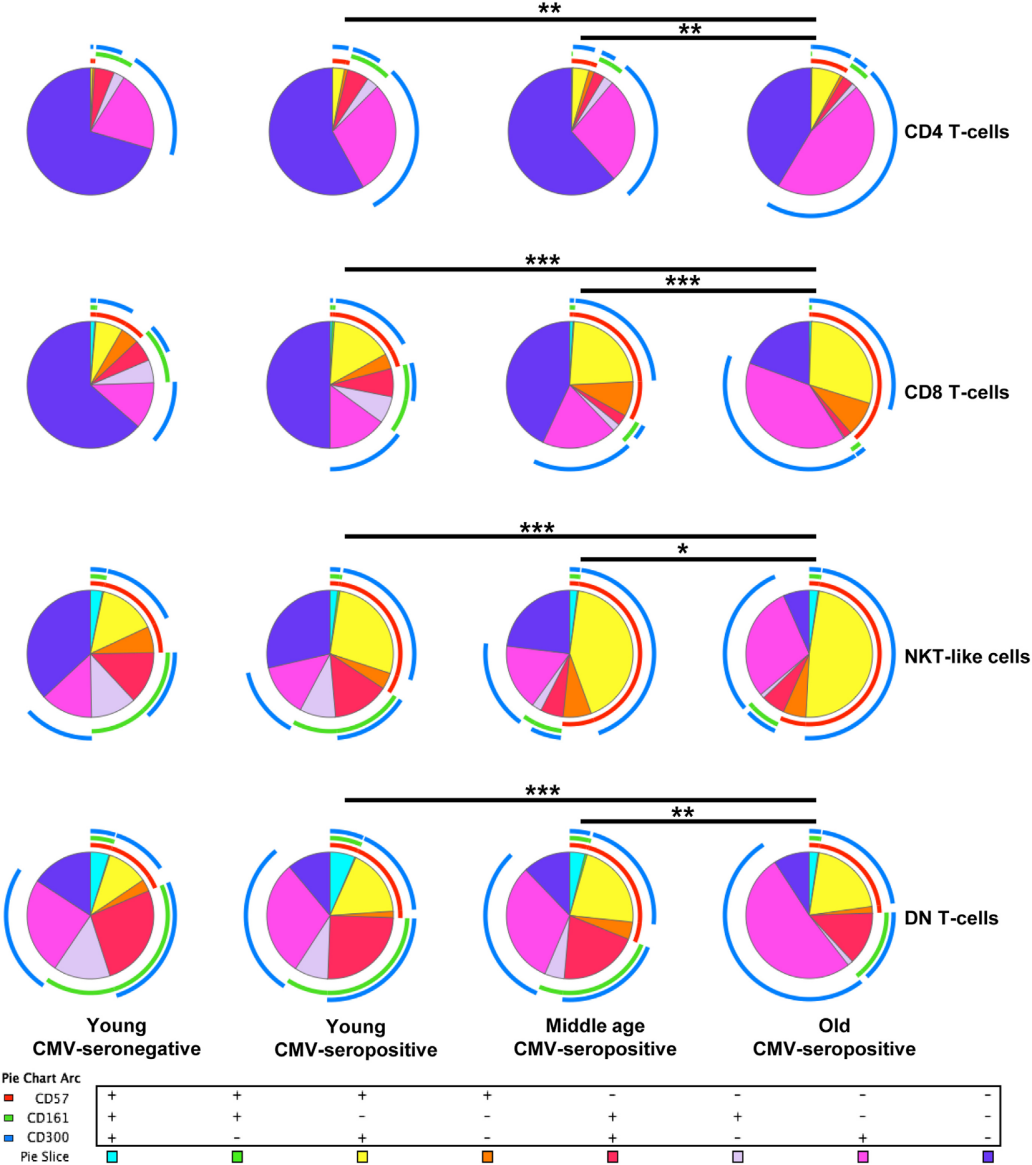
Expression of CD57, CD161, and CD300a in T-cells. CD57, CD161, and CD300a co-expression patterns (pie charts) in CD4^+^, CD8^+^, NKT-like, and DN T-cells from healthy individuals (*n* = 64), stratified by age and CMV serostatus. Results were considered significant at *p < 0.05, **p < 0.01, and ***p < 0.001.

FlowJo’s Boolean analysis of CD57, CD161, and CD300a expression generated eight different possible phenotype combinations per T-cell subset. However, not all the possible combinations were biologically meaningful. Phenotype profiles for each subset were obtained using SPICE software (Figure 1).

#### CD4^+^ T-Cells

**Figure 2:**
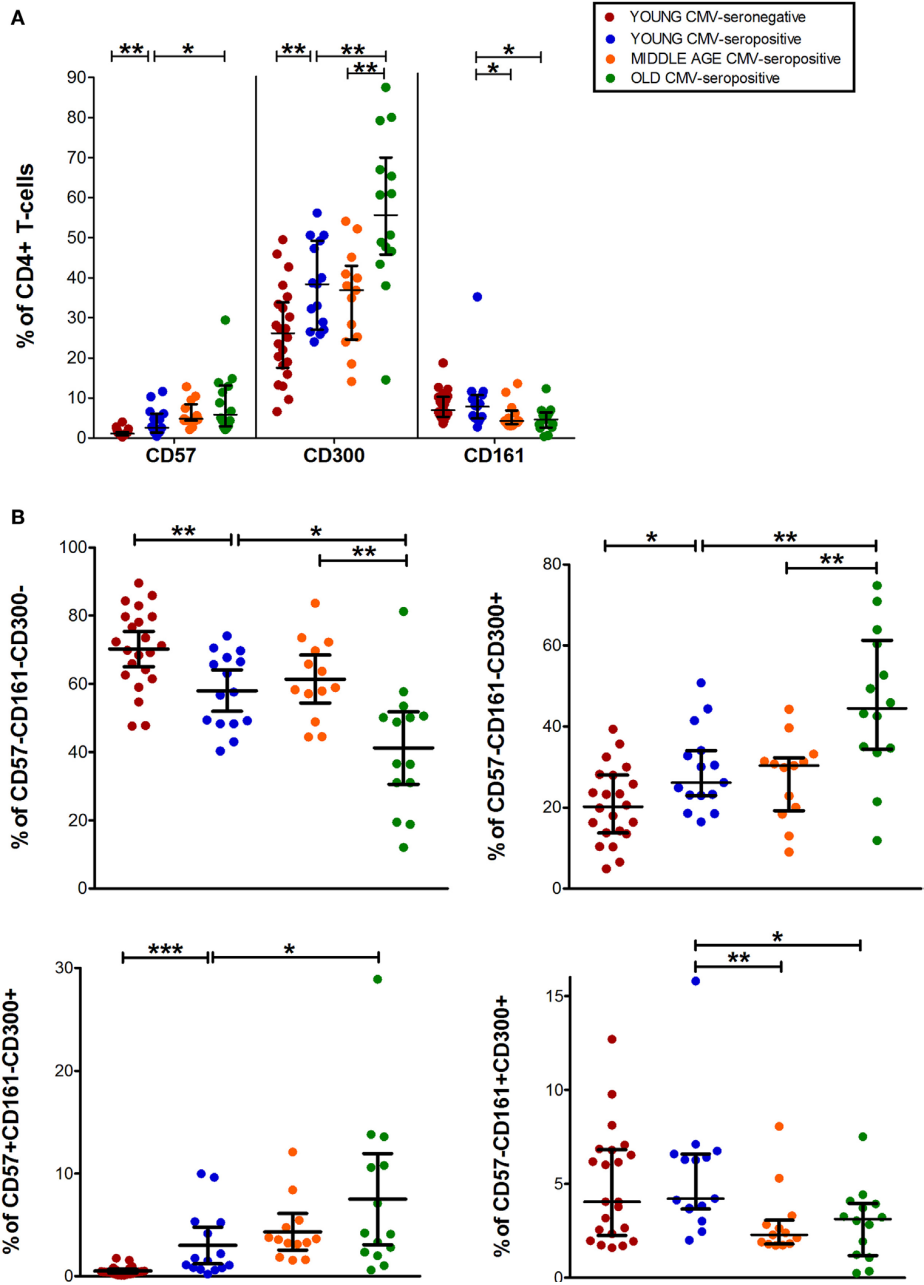
CD57, CD300, and CD161 expression on CD4^+^ T-cells. **(A)** Total expression (percentage) of CD57, CD161, and CD300a on CD4^+^ T-cells from young CMV-seronegative (*n* = 22), young CMV-seropositive (*n* = 15), middle age CMV-seropositive (*n* = 13), and old CMV-seropositive donors (*n* = 14). **(B)** Co-expression of CD57, CD161, and CD300a on CD4^+^ T-cells. Graphs show CD4^+^ T-cell phenotypes in which we found statistical differences among the four groups studied. Vertical blacklines indicate interquartile ranges, ranging from the 25th to the 75th percentile. The median values are indicated by a horizontal black line. Results were considered significant at *p < 0.05, **p < 0.01, and ***p < 0.001.

Analysis of CD4^+^ T-cell subset showed that CD57^+^CD4^+^ and CD300a^+^CD4^+^ T-cells increased with age in CMV-seropositive individuals and with CMV infection in young individuals (Figure 2A). In contrast, CD161^+^CD4^+^ T-cells percentage was decreased with age (Figure 2A).

Out of the eight possible Boolean phenotype combinations, we only found five within the CD4^+^ T-cell subset, as the percentages of cells with CD57^+^CD161^+^CD300a^+^, CD57^+^CD161^+^CD300a^−^ and CD57^+^CD161^−^CD300a^−^ phenotypes were noticeably low or null in all subjects studied (Figure 1).

The majority of CD4^+^ T-cells in young and middle-age individuals did not express any of the markers studied (CD57^−^CD161^−^CD300a^−^). However, in the elderly, more than 50% of the cells were CD300a^+^ (55.67%, IQR 46.62–66.99) alone or in combination with CD161 or CD57 (Figures 1 and 2A, Table S1 in Supplementary Material). Our data as well showed that the percentage of triple negative (CD57^−^CD161^−^CD300a^−^) CD4^+^ T-cells was decreased by CMV infection in young individuals. The progressive reduction of CD57^−^CD161^−^CD300a^−^ CD4^+^ T-cells by CMV infection and age corresponded with an increase of CD57^−^CD161^−^CD300a^+^ and CD57^+^CD161^−^CD300^+^ phenotypes (Figure 2B). Of note, CD57^+^CD4^+^ T-cells were only present in CMV-seropositive individuals and always co-expressing CD300a (Figures 1 and 2B).

On the other hand, our analysis showed that CD161 is never co-expressed with CD57 in any of the groups studied (Figure 1). CD57^−^CD161^+^CD300a^−^ and CD57^−^CD161^+^CD300a^+^CD4^+^ T-cells decreased with age in CMV-seropositive individuals, being the percentage of CD57^−^CD161^+^CD300a^−^ cells very low or null in the elderly (Figures 1 and 2B).

Furthermore, the phenotype profiles of CD4^+^ T-cells changed with age in CMV-seropositive individuals, but not with CMV infection alone (pie charts representing the three makers’ combinations, Figure 1). This shift of phenotype is mainly due to an accumulation with age of the CD57^−^CD161^−^CD300a^+^ and CD57^+^CD161^−^CD300a^+^ phenotypes in the CMV-seropositive individuals.

#### CD8^+^ T-Cells

**Figure 3:**
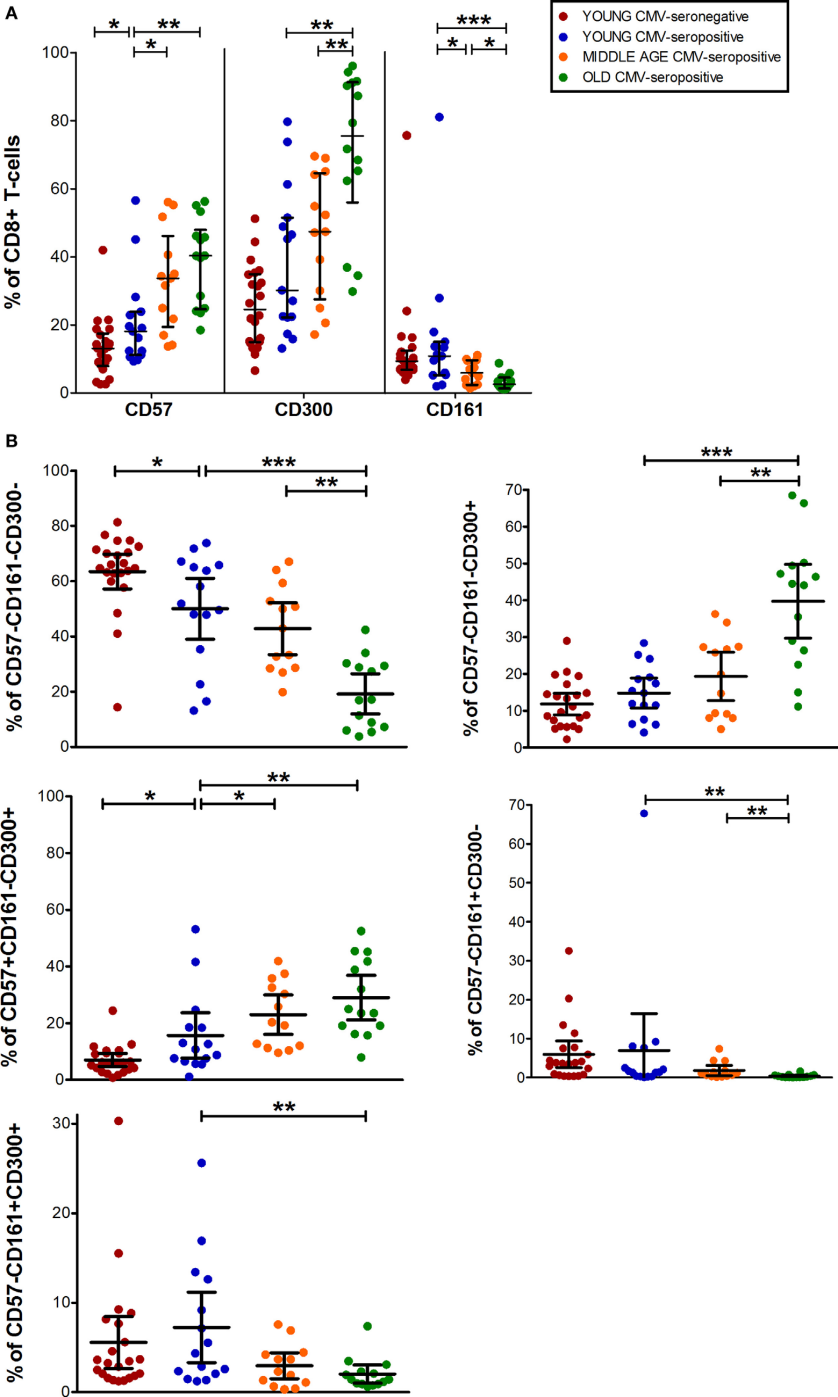
CD57, CD300, and CD161 expression on CD8^+^ T-cells. **(A)** Total expression (percentage) of CD57, CD161, and CD300a markers on CD8^+^ T-cells from young CMV-seronegative (*n* = 22), young CMV-seropositive (*n* = 15), middle age CMV-seropositive (*n* = 13), and old CMV-seropositive donors (*n* = 14). **(B)** Co-expression of CD57, CD161, and CD300a on CD8^+^ T-cells. Graphs show the phenotype combinations CD8^+^ T-cells in which we found statistical differences among the four groups studied. Vertical blacklines indicate interquartile ranges, ranging from the 25th to the 75th percentile. The median values are indicated by a horizontal black line. Results were considered significant at *p < 0.05, **p < 0.01, and ***p < 0.001.

Data from CD8^+^ T-cell subset showed that CD57^+^CD8^+^ T-cells increased with CMV infection alone and in combination with age. While, CD300a^+^CD8^+^ T-cells accumulate with age in CMV-seropositive individuals and CD161^+^CD8^+^ T-cells decreased progressively being very low or null in the elderly (Figures 1 and 3A).

The percentages of CD57^+^CD161^+^CD300a^+^ and CD57^+^CD161^+^CD300a^−^ CD8^+^ T-cells were noticeably low or null in all subjects studied. The majority of CD57^+^CD8^+^ T-cells were positive for CD300a and negative for CD161 (Figure 1). However, in contrast to CD4^+^ T-cells, in the CD8^+^ subset, we found a small fraction of cells with CD57^+^CD161^−^CD300a^−^ phenotype (pie slice orange, Figure 1), not affected by age.

In young and middle age individuals, 60–70% of the CD8^+^ T-cells were mainly CD57^−^CD161^−^CD300a^−^ (Table S1 in Supplementary Material). However, in the elderly, only 17% (IQR 7.20–29.30) of CD8^+^ T-cells did not express any of the markers (Figure 3B; Table S1 in Supplementary Material). This drastic reduction observed in the elderly is due to the expansion of CD300a^+^ cells with or without CD57 (yellow and pink pie slices, Figure 1). In young individuals, CD57^−^CD161^−^CD300a^−^ CD8^+^ T-cells decreased with CMV infection (Figure 3B) due to the expansion of CD57^+^CD161^−^CD300a^+^ cells (yellow pie slice, Figures 1 and 3B).

Additionally, we observed that in young and middle age individuals, CD161^+^CD8^+^ T-cells were CD300a^+^ or CD300a^−^, whereas in the elderly, the few CD161^+^ cells observed were all CD300a^+^ (pie slices red and violet, Figure 1).

The phenotype profiles (pie charts, Figure 1) of CD8^+^ T-cells changed noticeably with age in CMV-seropositive individuals, but not with CMV infection alone (Figure 1).

#### CD8^+^CD56^+^ T-Cells (NKT-Like Cells)

**Figure 4:**
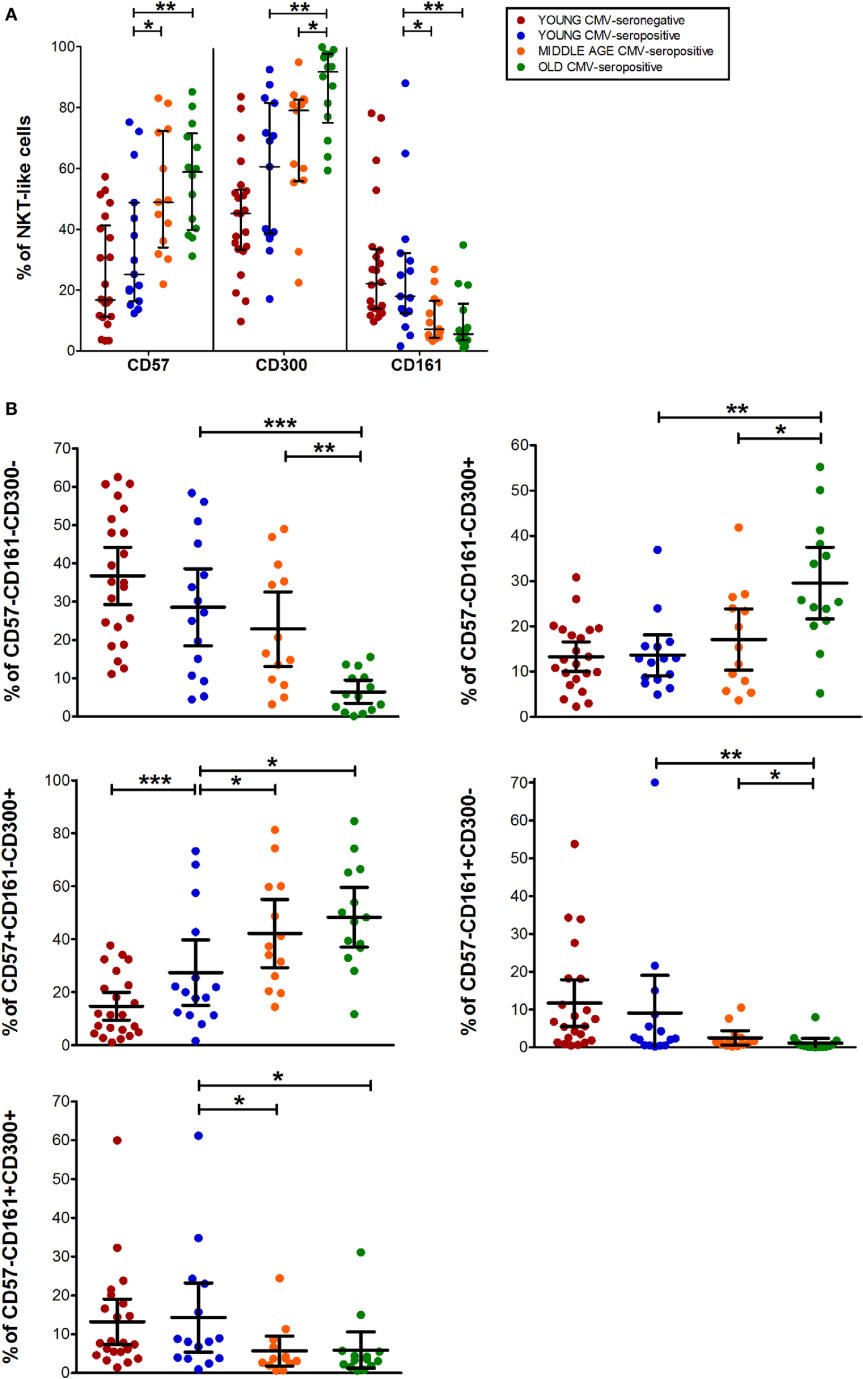
CD57, CD300, and CD161 expression on NTK-like cells. **(A)** Total expression (percentage) of CD57, CD161, and CD300a markers on NKT-like cells from young CMV-seronegative (*n* = 22), young CMV-seropositive (*n* = 15), middle age CMV-seropositive (*n* = 13), and old CMV-seropositive donors (*n* = 14). **(B)** Co-expression of CD57, CD161, and CD300a on NKT-Like cells. Graphs show the markers combinations in which we found statistical differences among the four groups studied. Vertical black lines indicate interquartile ranges, ranging from the 25th to the 75th percentile. The median values are indicated by a horizontal black line. Results were considered significant at *p < 0.05, **p < 0.01, and ***p < 0.001.

The expression of CD57, CD161, and CD300a markers on NKT-like cells was not affected by CMV infection alone. However, CD57^+^ and CD300a^+^ NKT-like cells increased with age in CMV-seropositive individuals (Figure 4A), while CD161^+^ NKT-like cells decreased (Figure 4A).

CD57^+^CD161^+^CD300a^+^ and CD57^+^CD161^+^CD300a^−^ NKT-like cells were very low or null. Thus, in our hands, as CD4^+^ and CD8^+^ subsets, NKT-like cells did not co-express CD57 and CD161 (pie slice orange, Figure 1). The majority of CD57^+^ NKT-like cells were also CD300a^+^. However, we observed a small fraction of NKT-like cells with a CD57^+^CD161^−^CD300a^−^ phenotype, not affected by CMV infection and age (Figure 1). Of note, in the elderly, 47.35% (IQR 36.70–65.10) of the NKT-like cells were CD57^+^CD161^−^CD300a^+^ (yellow pie slice, Figure 1). The proportion of this phenotype is significantly lower (*p* < 0.001) in the rest of the T-cell subsets studied, particularly in the CD4^+^ T-cells in which this phenotype frequency is quite low even in the elderly (Figure 1).

In the NKT-like subset, 65–95% of the cells expressed at least one of the markers studied, being the fraction of triple negative cells (CD57^−^CD161^−^CD300a^−^) very low in old individuals (5.58%, IQR 1.77–10.20) (Figures 1 and 4B), due to the expansion of CD57^−^CD161^−^CD300a^+^ and CD57^+^CD161^−^CD300a^+^ phenotypes (pie slices pink and yellow, Figure 1). Noticeably, CD57^+^CD161^−^CD300a^+^ cells were also increased in young CMV-seropositive individuals compared with CMV-seronegative (Figure 4B).

NKT-like CD161^+^ phenotypes (CD57^−^CD161^+^CD300a^−^ and CD57^−^CD161^+^CD300a^+^) decreased with age in CMV-seropositive individuals, but not with CMV infection alone (Figures 1 and 4B). Remarkably, the percentage of CD57^−^CD161^+^CD300a^−^ NKT-like cells was very low or null in middle age and old individuals (Figure 4B; Table S1 in Supplementary Material).

As in the CD4^+^ and CD8^+^ main populations, the phenotype profiles of NKT-like cells were not affected by CMV infection alone (Figure 1).

#### CD4^−^CD8^−^ T-Cells (DN T-Cells)

**Figure 5:**
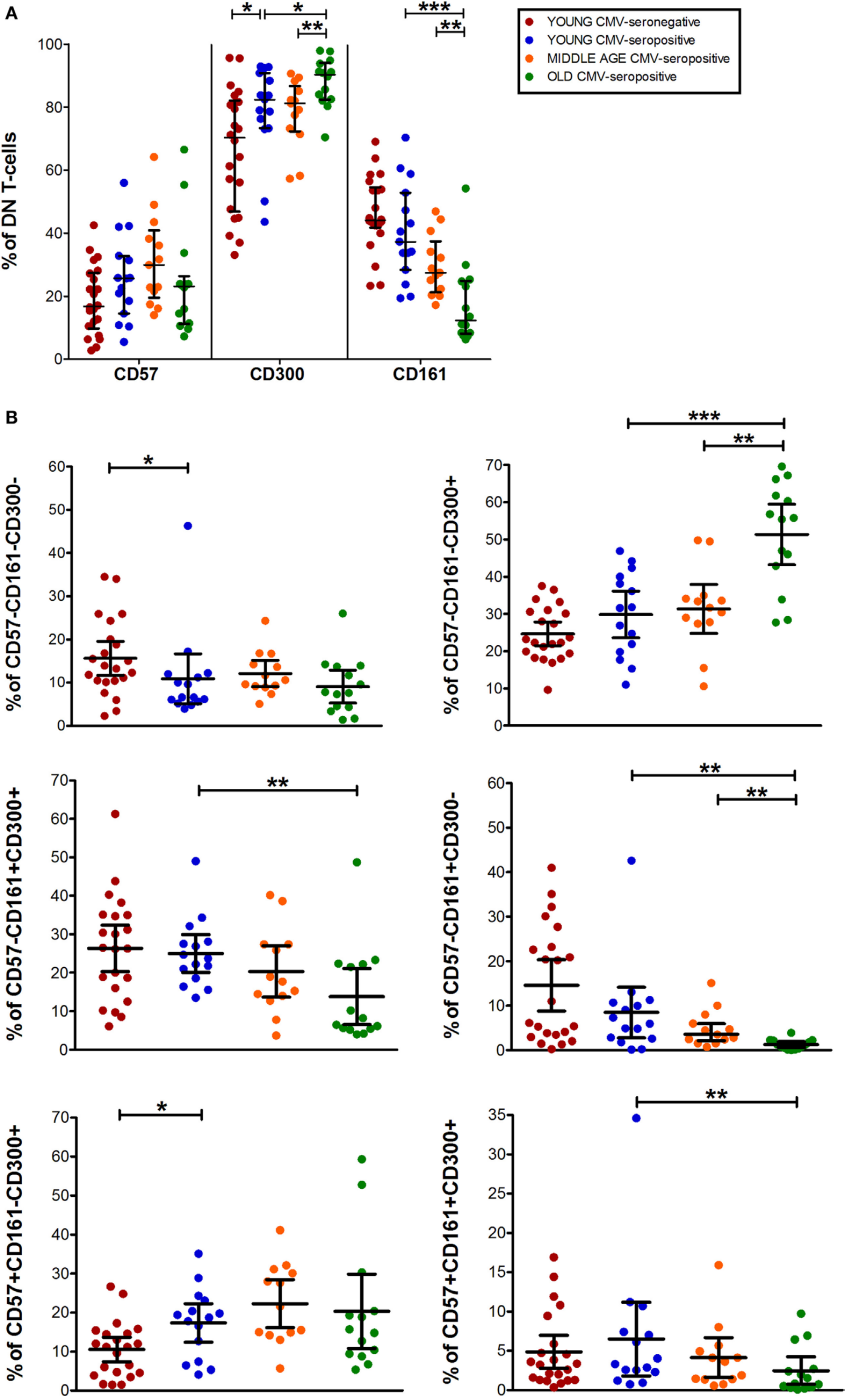
CD57, CD300, and CD161 expression on CD4^−^CD8^−^ T-cells (DN). **(A)** Total expression (percentage) of CD57, CD161, and CD300a markers on DN T-cells from young CMV-seronegative (*n* = 22), young CMV-seropositive (*n* = 15), middle age CMV-seropositive (*n* = 13), and old CMV-seropositive donors (*n* = 14). **(B)** Coexpression of CD57, CD161, and CD300a on DN T-cells. Graphs show DN T-cells phenotypes in which we found statistical differences among the four groups studied. Vertical black lines indicate interquartile ranges, ranging from the 25th to the 75th percentile. The median values are indicated by a horizontal black line. Results were considered significant at *p < 0.05, **p < 0.01, and ***p < 0.001.

Data from DN T-cells (majorly γδ T-cells) flow analysis showed a similar percentage of CD57^+^ DN T-cells among the three CMV-seropositive groups (young, middle age, and old) (Figure 5A). While, CD161^+^ DN T-cell decreased gradually with age in CMV-seropositive individuals (Figure 5A). Furthermore, CD300a^+^ DN T-cells increased with CMV infection in young individuals and further increased in old CMV-seropositive individuals (Figure 5A).

As in the other T-cell subsets studied, in DN T-cells the percentage of CD57^+^CD161^+^CD300a^−^ cells is null (Figure 1). However, we observed a small fraction of DN T-cells co-expressing the three markers that decreases with age (Figures 1 and 5B).

The majority of DN T-cells in all individuals are CD300a^+^ with or without CD161 or CD57 expression (Figure 1). Our analysis showed that the percentage of CD57^−^CD161^−^CD300a^+^ cells increased with age in CMV-seropositive individuals, whereas the percentage of CD57^+^CD161^−^CD300a^+^ increases with CMV infection in young individuals and is not affected by age (Figure 5B). Of note, our results showed that, similarly to CD57^+^CD4^+^ T-cells, the majority of CD57^+^ DN T-cells are as well CD300^+^ (Figure 1).

Besides, CD161^+^ DN T-cells were mainly CD57^−^ (only a small fraction co-expressed CD161 and CD57). CD57^−^CD161^+^CD300a^−^ phenotype decreased with age, being the percentage of these cells null in the elderly (pie slice violet, Figures 1 and 5B). Whereas a reduced percentage of CD57^−^CD161^+^CD300a^+^ DN T-cells was still present in old individuals (pie slice red, Figures 1 and 5B).

DN T-cell phenotype profiles for the makers studied (pie charts, Figure 1) changed with age in CMV-seropositive individuals, but not with CMV infection alone.

## Discussion

The combination of age and CMV latent infection has been proven to have a profound impact on the immune phenotype and function of T-cells, not only on the CD8^+^ subset but also on CD4^+^, NKT-like, and γδ T-cells. However, age and CMV infection do not always have similar effects and it can vary depending on the cell type.

Here, we analyzed, in different T-cell subsets, how age and CMV infection alter the expression of the inhibitory receptors CD300a and CD161 and their relation with the marker CD57, which has been shown to be a polyfunctionality maker of CD4^+^, CD8^+^, and NKT-like T-cells (37, 38, 49). We are aware that due to the high prevalence of CMV in our geographic area (see Materials and Methods), a limitation of our study is the lack of CMV-seronegative individuals of older ages (middle age and old groups). Thus, we can only assess the effect of aging in the context of CMV latent infection. Nevertheless, we were able to investigate the effect of CMV infection alone in young individuals.

Our analysis showed that in all T-cell subsets studied, CD57 and CD300a increase with age in CMV-seropositive individuals. Specifically, with CMV infection (young individuals), CD57 is increased only in CD4^+^ and CD8^+^ T-cells and CD300a in CD4^+^ and DN subsets. No effect of CMV alone was observed on NKT-like cells. Of note, CD57^+^CD4^+^ T-cells are always CD300a^+^ and were only found in CMV-seropositive individuals. In the rest of subsets (CD8^+^, NKT-like and DN), although not all, the majority of CD57^+^ T-cells were CD300a^+^ as well, regardless of the age and CMV serostatus.

The expression of CD300a by several immune cell types has been associated with different pathologies, suggesting that, although the significance of CD300a on T-cell function is not completely clear, CD300a could be used as a biomarker and a target for new therapies [for review, see Ref. (50)].

CD300a ligands, phosphatidylserine (PS), and phosphatidylethanolamine (PE) are associated with virus evasion. Indeed, anti-PS antibody has been shown to be a potential treatment for CMV and Pichinde virus infections (51). Additionally, HIV-specific CD8^+^ T-cell mRNA levels of CD300a receptor have been shown to correlate with the expression of BATF transcription factor, which is highly expressed in exhausted cells. BTAF inhibits cell function by inducing the expression of inhibitory receptors such as CD300a (52). Viral envelope expression of PS and PE has been shown to be an evasion mechanism called “apoptotic mimicry” (53). However, CD300a binding to viruses expressing PS and PE in their envelopes seams to inhibit the virus endocytosis, most probably hampering the virus infection. All these data supports that CD300a has an inhibitory role and it is important for viral infections.

However, in CD8^+^ T-cells, CD300a expression associates with higher cytotoxicity and CD300a^+^CD8^+^ T-cells are increased in pregnant women with chronic choriamnionitis (10). Additionally, CD300a has been shown to be a polyfunctionality marker in CD4^+^ T-cells and CD300a^+^CD4^+^ T-cells upregulate Eomes transcription factor after stimulation (11). Furthermore, our group has recently shown that CD57^+^CD4^+^ T-cells are polyfunctional and express high levels of T-bet and Eomes transcription factors upon superantigen stimulation (38). Moreover, CD57^+^CD8^+^ T-cells correlate with polyfunctionality of CD8^+^ T-cells and are expanded in young CMV-seropositive individuals (37).

Whether CD300a^+^CD57^−^ and CD300a^+^CD57^+^ T-cells display any differences regarding polyfunctionality and if there are differences in regards of T-bet and Eomes expression is currently under investigation in our laboratory. This analysis will allow us to establish if CD300a is a polyfunctional marker of T-cells *per se*, or only if co-expressed with CD57. In our hands CD57^+^ T-cells co-expressing CD300a expand with CMV infection (in young individuals), highlighting a relevant role for both makers in the control of CMV virus by T-cells. On the other hand, CD161 receptor was hardly co-expressed with CD57 in any of the T-cell subsets studied. Particularly, co-expression of CD161 and CD57 was not observed in the elderly regardless of the T-cell subset. Furthermore, the total expression of CD161, contrarily to CD57 and CD300a, decreases with age in CMV-seropositive individuals and is not affected by CMV infection alone in young individuals. Our results support previous results from healthy children in which the expression of CD161 on T and NK cells was not affected by CMV serostatus (54). However, Almehmadi et al. suggest that NKT-like cells not expressing CD161 are increased in CMV-seropositive individuals. This discrepancy with our data can be explained by the fact that Almehmadi’s cohort does not stratify the individuals by age (23–60 years), only by CMV status. Additionally, their definition of NKT-like cells differs from ours as it is based only on CD3 and CD56 expression, not including CD8 [for review of NKT-like cells nomenclatures, see Ref. (55)]. In our previous work, regarding NKT-like cell number and functionality in the context of CMV infection and age, we show that the percentage of NKT-like cells is not affected by CMV infection in young CMV individuals, but rather with the combined effect of both age and CMV latent infection (49). Similarly, the loss of CD161 by T-cells does not associate with CMV alone, but with age in the context of CMV latent infection. Indeed, the expression of CD161 in CMV-specific cytotoxic T lymphocytes is very low (56).

Besides, acute and chronic GVHD correlates with decreased levels of circulating CD161^+^CD4^+^ and CD161^++^CD8^+^ T-cells (57). Moreover, in rheumatoid arthritis patients, it has been shown an increase of CD161^+^CD4^+^ T-cells, but a decrease of CD161^+^ DN T-cells that was associated with disease activity and inflammation (58, 59). As we mentioned before, T-cells-expressing CD161 are IL-17 producers. In our cohort, the percentages of CD161^+^ T-cells are very low or null in old CMV-seropositive individuals regardless of the T-cell type. This could translate in a diminished Th17 response in the elderly.

Contrarily to what we observe in T-cells, our previous work on NK cells showed a decreased expression of CD161 on CD56dim NK cells associated with CMV seropositivity (60).

The data presented here together with our previous results highlight the importance of taking into account both age and CMV serostatus in any clinical study regarding the analysis of T-cells, as CMV latent infection has a differential effect with age on T-cell subsets. Additionally, our data support the potential use of CD57, CD300a, and CD161 as biomarkers of immunosenescence and as possible targets for novel therapies. The clinical implications of the changes found in the expression of these makers should be further investigated.

## Ethics Statement

This work was approved by the Ethics Committee of the Reina Sofia University Hospital. All participants in the study provided informed written consent.

## Author Contributions

RS and AP designed the study. FH collected the data and performed the laboratory experiments. FH and NL-S collaborated in the laboratory analysis. AP and FH performed the statistical analysis and wrote the draft. RT, BS-C, and CC made significant technical and conceptual contributions to the manuscript. RS, RT, and AP reviewed the final version of the paper. All the authors provided intellectual content and approved the final version of the paper. RS an AP are co-senior authors and have contributed equally to this work.

## Conflict of Interest Statement

The authors declare that the research was conducted in the absence of any commercial or financial relationships that could be construed as a potential conflict of interest.
