# Supplementary material for: Differential Effect of Cytomegalovirus Infection with Age on the Expression of CD57, CD300a, and CD161 on T-Cell Subpopulations
Source: Front Immunol. 2017 Jun 2;8:649. doi: 10.3389/fimmu.2017.00649 (PMC5454039; doi:10.3389/fimmu.2017.00649)
Supplement: Supplementary file 1 [file Table_1.PDF]

Table S1. Median and InterQuartile Range (IQR) of the Co-expression analysis of CD57, CD300a and CD161 on T-cell subsets A) CD4 and CD8 phenotypes

CMV status	CD4+ phenotype	Median	Q1 Percentile 25	Q3 Percentile 75	CD8+ phenotype	Median	Q1 Percentile 25	Q3 Percentile 75
Young CMV-seronegative	CD4+CD57+CD161+CD300+	.07	.04	.11	CD8+CD57+CD161+CD300+	.49	.21	.72
	CD4+CD57+CD161+CD300-	.03	.02	.04	CD8+CD57+CD161+CD300-	.16	.06	.28
	CD4+CD57+CD161-CD300+	.41	.22	.60	CD8+CD57+CD161-CD300+	5.94	3.66	9.37
	CD4+CD57+CD161-CD300-	.49	.40	.69	CD8+CD57+CD161-CD300-	2.98	1.28	7.60
	CD4+CD57-CD161+CD300+	4.05	2.35	6.80	CD8+CD57-CD161+CD300+	3.34	1.81	7.64
	CD4+CD57-CD161+CD300-	2.73	2.33	3.62	CD8+CD57-CD161+CD300-	3.69	.76	7.50
	CD4+CD57-CD161-CD300+	20.25	13.80	28.00	CD8+CD57-CD161-CD300+	10.42	5.79	14.80
	CD4+CD57-CD161-CD300-	70.50	62.60	79.70	CD8+CD57-CD161-CD300-	65.30	62.80	71.40
Young CMV-seropositive	CD4+CD57+CD161+CD300+	.10	.05	.33	CD8+CD57+CD161+CD300+	.70	.35	.96
	CD4+CD57+CD161+CD300-	.02	.01	.06	CD8+CD57+CD161+CD300-	.08	.03	.21
	CD4+CD57+CD161-CD300+	1.51	.83	5.23	CD8+CD57+CD161-CD300+	10.70	6.48	18.50
	CD4+CD57+CD161-CD300-	.55	.36	.64	CD8+CD57+CD161-CD300-	3.25	1.48	5.58
	CD4+CD57-CD161+CD300+	4.22	3.66	6.59	CD8+CD57-CD161+CD300+	4.33	2.05	12.60
	CD4+CD57-CD161+CD300-	2.11	1.05	4.05	CD8+CD57-CD161+CD300-	1.40	.29	7.68
	CD4+CD57-CD161-CD300+	26.20	23.00	34.10	CD8+CD57-CD161-CD300+	14.80	7.61	19.10
	CD4+CD57-CD161-CD300-	57.60	48.30	67.70	CD8+CD57-CD161-CD300-	51.80	35.30	65.80
Middle age CMV-seropositive	CD4+CD57+CD161+CD300+	.23	.07	.33	CD8+CD57+CD161+CD300+	.60	.35	.96
	CD4+CD57+CD161+CD300-	.02	.02	.04	CD8+CD57+CD161+CD300-	.16	.04	.42
	CD4+CD57+CD161-CD300+	3.57	3.12	4.77	CD8+CD57+CD161-CD300+	20.30	12.00	32.50
	CD4+CD57+CD161-CD300-	.70	.63	1.11	CD8+CD57+CD161-CD300-	7.34	2.17	11.60
	CD4+CD57-CD161+CD300+	2.29	1.82	2.83	CD8+CD57-CD161+CD300+	2.28	1.08	4.19
	CD4+CD57-CD161+CD300-	1.72	1.22	2.30	CD8+CD57-CD161+CD300-	1.10	.39	1.79
	CD4+CD57-CD161-CD300+	30.40	20.10	31.40	CD8+CD57-CD161-CD300+	19.90	9.14	27.30
	CD4+CD57-CD161-CD300-	58.90	57.10	69.70	CD8+CD57-CD161-CD300-	42.90	28.60	52.70
Old CMV-seropositve	CD4+CD57+CD161+CD300+	.13	.08	.40	CD8+CD57+CD161+CD300+	.39	.30	.82
	CD4+CD57+CD161+CD300-	.02	.01	.04	CD8+CD57+CD161+CD300-	.03	.00	.09
	CD4+CD57+CD161-CD300+	4.15	2.36	10.80	CD8+CD57+CD161-CD300+	24.25	19.10	41.80
	CD4+CD57+CD161-CD300-	.71	.42	1.33	CD8+CD57+CD161-CD300-	2.68	1.14	11.10
	CD4+CD57-CD161+CD300+	3.14	1.23	3.93	CD8+CD57-CD161+CD300+	1.42	.91	2.28
	CD4+CD57-CD161+CD300-	1.15	.55	2.52	CD8+CD57-CD161+CD300-	.22	.07	.61
	CD4+CD57-CD161-CD300+	44.55	34.70	60.40	CD8+CD57-CD161-CD300+	44.30	26.40	49.40

Table S1. Median and InterQuartile Range (IQR) of the Co-expression analysis of CD57, CD300a and CD161 on T-cells subsets B) NKT-like and DN phenotypes

CMV status	NKT-like phenotype	Median	Q1 Percentile 25	Q3 Percentile 75	DN phenotype	Median	Q1 Percentile 25	Q3 Percentile 75
Young CMV-seronegative	CD8+CD56+CD57+CD161+CD	1.20	.56	3.79	CD4-CD8-	3.29	1.29	5.88
	CD8+CD56+CD57+CD161+CD	.26	.06	.82	CD4-CD8-	.19	.05	.52
	CD8+CD56+CD57+CD161-	11.40	4.91	22.60	CD4-CD8-CD57+CD161-	11.20	4.53	14.60
	CD8+CD56+CD57+CD161-	2.44	1.15	10.20	CD4-CD8-CD57+CD161-	2.15	1.00	3.77
	CD8+CD56+CD57-	7.72	5.42	17.90	CD4-CD8-CD57-	26.40	16.00	35.00
	CD8+CD56+CD57-	6.15	1.83	18.10	CD4-CD8-CD57-	8.56	3.48	23.20
	CD8+CD56+CD57-CD161-	12.20	8.37	19.20	CD4-CD8-CD57-CD161-	22.75	19.40	30.60
	CD8+CD56+CD57-CD161-	35.10	23.40	51.60	CD4-CD8-CD57-CD161-CD300-	13.55	10.40	20.00
Young CMV-seropositive	CD8+CD56+CD57+CD161+CD	1.34	1.00	2.50	CD4-CD8-	3.31	2.29	7.41
	CD8+CD56+CD57+CD161+CD	.17	.02	.50	CD4-CD8-	.07	.03	.40
	CD8+CD56+CD57+CD161-	20.00	11.20	42.70	CD4-CD8-CD57+CD161-	18.70	7.43	23.10
	CD8+CD56+CD57+CD161-	3.03	.94	6.19	CD4-CD8-CD57+CD161-	1.66	.68	2.19
	CD8+CD56+CD57-	8.09	3.77	23.00	CD4-CD8-CD57-	23.70	18.60	28.10
	CD8+CD56+CD57-	2.32	.50	8.69	CD4-CD8-CD57-	5.94	2.60	10.70
	CD8+CD56+CD57-CD161-	12.90	8.27	15.60	CD4-CD8-CD57-CD161-	31.60	19.80	40.00
	CD8+CD56+CD57-CD161-	27.20	10.70	45.20	CD4-CD8-CD57-CD161-CD300-	6.59	5.65	12.00
Middle age CMV-seropositive	CD8+CD56+CD57+CD161+CD	1.24	.93	1.65	CD4-CD8-	3.62	1.40	4.93
	CD8+CD56+CD57+CD161+CD	.19	.05	.29	CD4-CD8-	.21	.02	.55
	CD8+CD56+CD57+CD161-	37.30	26.00	59.70	CD4-CD8-CD57+CD161-	21.60	14.90	30.10
	CD8+CD56+CD57+CD161-	5.16	2.12	10.20	CD4-CD8-CD57+CD161-	3.02	1.70	4.89
	CD8+CD56+CD57-	3.67	2.48	6.65	CD4-CD8-CD57-	17.70	14.00	27.40
	CD8+CD56+CD57-	1.40	.71	2.54	CD4-CD8-CD57-	3.52	2.42	5.99
	CD8+CD56+CD57-CD161-	15.40	7.95	24.00	CD4-CD8-CD57-CD161-	31.70	27.80	34.10
	CD8+CD56+CD57-CD161-	16.50	9.75	35.30	CD4-CD8-CD57-CD161-CD300-	10.60	9.16	14.20
old CMV-seropositve	CD8+CD56+CD57+CD161+CD	1.15	.87	2.75	CD4-CD8-	1.19	.37	2.70
	CD8+CD56+CD57+CD161+CD	.01	.00	.10	CD4-CD8-	.00	.00	.02
	CD8+CD56+CD57+CD161-	47.35	36.70	65.10	CD4-CD8-CD57+CD161-	15.25	9.42	20.40
	CD8+CD56+CD57+CD161-	2.07	.58	8.92	CD4-CD8-CD57+CD161-	1.06	.70	2.68
	CD8+CD56+CD57-	3.40	1.82	5.47	CD4-CD8-CD57-	7.35	5.46	22.20
	CD8+CD56+CD57-	.34	.10	1.08	CD4-CD8-CD57-	1.05	.33	2.17
	CD8+CD56+CD57-CD161-	25.60	21.30	38.20	CD4-CD8-CD57-CD161-	55.60	42.90	61.80
