# Supplementary material for: Differential Effect of Cytomegalovirus Infection with Age on the Expression of CD57, CD300a, and CD161 on T-Cell Subpopulations
Source: Front Immunol. 2017 Jun 2;8:649. doi: 10.3389/fimmu.2017.00649 (PMC5454039; doi:10.3389/fimmu.2017.00649)
Supplement: Supplementary file 2 [file Image_1.PDF]

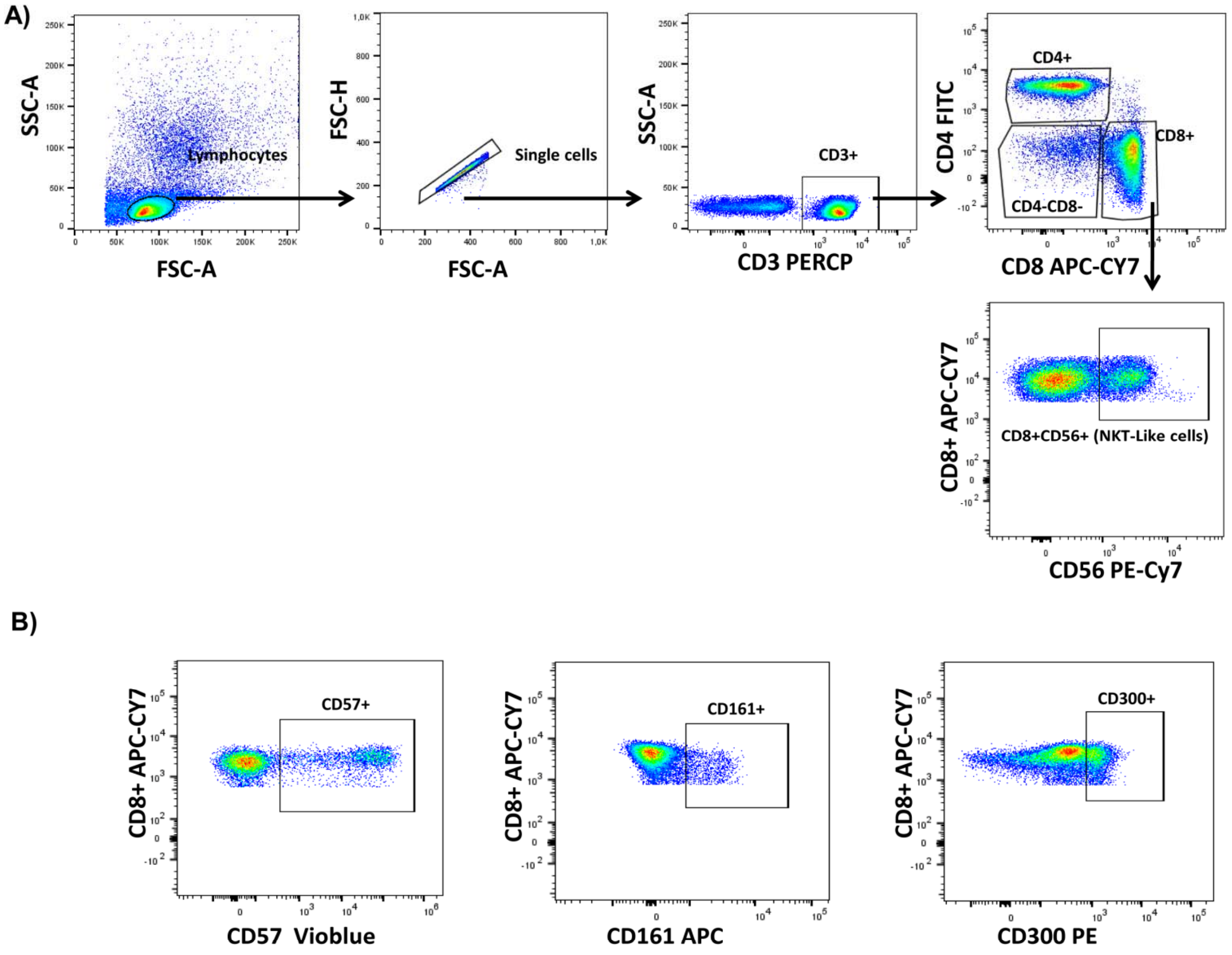

Figure S1. Gating strategy used for the analysis of CD57, CD161 and CD300a expression on T-cells. A) Peripheral blood lymphocytes (PBLs) were selected using forward (FSC) and side scatter (SSC) detectors, subsequently CD3+ T-cells were gated from PBLs after single cells gating , followed by identification of CD4+, CD8+, CD4–CD8– T-cells (DN: Double negative) and CD8+CD56+ T-cells (NKT-Like cells). B) Total expression of CD57, CD161 and CD300a was determined within each T-cell subset using Fluorescence minus one (FMO) controls. Figure shows an example for CD8+ T-cell subset.
